# Trait-based characterisation of soil exploitation strategies of banana, weeds and cover plant species

**DOI:** 10.1371/journal.pone.0173066

**Published:** 2017-03-03

**Authors:** Florence Tardy, Gaëlle Damour, Marc Dorel, Delphine Moreau

**Affiliations:** 1 CIRAD, UPR GECO, Capesterre-Belle-Eau, Guadeloupe, France; 2 Agroécologie, AgroSup Dijon, INRA, Univ. Bourgogne Franche-Comté, Dijon, France; Instituto Agricultura Sostenible, SPAIN

## Abstract

Cover plants can be introduced in cropping systems to provide agroecosystem services, including weed control via competition for resources. There is currently no consensus on how to identify the best cover plant species, while trait-based approaches are promising for screening plant species due to their agroecosystem service provision potential. This study was carried out to characterize soil exploitation strategies of cover plant species in banana agroecosystems using a trait-based approach, and in turn identify cover plant species with a high weed control potential via competition for soil resources in banana cropping systems. A field experiment was conducted on 17 cover plant species, two weed species and two banana cultivars grown individually. Four functional traits were measured. Two of them (i.e., the size of the zone explored by roots and the root impact density) were used to characterize root system soil exploration patterns. Two other traits (i.e., specific root length and root diameter) were used to characterize resource acquisition within the soil zone explored by the roots. All studied traits exhibited marked variations among species. The findings suggested a trade-off between the abilities of species to develop a limited number of large diameter roots exploring a large soil zone versus many thin roots exploring a smaller soil zone. Three soil-resource exploitation strategies were identified among species: (i) with large diameter roots that explore a large soil zone; (ii) with small diameter roots and a high specific length that explore a smaller soil zone; and (iii) with a high total root-impact density and an intermediate specific root length that explore the uppermost soil layers. Interestingly, in our panel of species, no correlations with regard to belowground and aboveground strategies were noted: species with an acquisitive belowground strategy could display an acquisitive or a conservative aboveground strategy. The findings of this study illustrated that a trait-based approach could be used to identify plant species with potential for competing with weeds, while minimising competition with banana. Six of the 17 studied cover crop species were identified as having this potential. The next step will be to assess them for their weed control performances in banana cropping systems with low reliance on herbicides.

## Introduction

Cover plants can provide agroecosystem services. For instance, they may improve resource use efficiency or control soil erosion and pests [1–3]. They can be grown during the fallow period or in association with the cash crop to control weeds through resource competition, thereby reducing reliance on herbicides. The effectiveness of biological control of pests such as weeds varies with the cover plant species [3–5]. However, to date, no tools are available to identify the most suitable cover plant species according to the targeted services.

Weed control by cover plants relies mainly on competition for available resources between the cover plant and the weed species. When choosing a cover plant for an association with the cash crop, the challenge is to maximise competition with weeds while minimising competition with the cash crop so as to avoid negative impacts on crop growth and yield. There can be aboveground competition between species for light and/or belowground competition for water and nutrients. In agroecosystems with a tall cash crop such as banana, the cash crop:weed competition for light is limited to a short period after crop planting, when the cash crop plants are still small. Belowground competition occurs throughout the crop cycle, probably because cash crop and weed plants share a similar spatial niche in the soil. This suggests that cover plants cannot be used to compete with weeds for soil resources without also generating competition with banana for the same resources. Weed control using cover plants should aim to maximise competition with weeds while minimising competition with banana. This could be achieved using cover plants with above- and belowground spatial niches that differ from those of banana [6], potentially leading to more complete use of resources. Aboveground complementarity between cover plants and cash crops could be achieved by choosing cover plants that are smaller than banana plants. Belowground complementarity could be achieved if the roots of both crop species explore different soil zones [7].

Trait-based approaches, originally developed in the field of comparative functional ecology, are a promising way to screen plant species for their ability to provide ecosystem services [8–10]. They rely on functional traits, i.e. the morphological, physiological and phenological features of individual plants that affect their performance [11]. Traits are related to plant-driven processes and can be used to compare a large number of species (e.g. [12]). Each species can be characterised by a combination of trait values to define its strategy, e.g. the manner in which a species secures carbon profit during vegetative growth and ensures future gene transmission [13]. Plant strategies can be characterized for specific processes (e.g. reproductive strategies in [14]) or at a more global scale via the so-called “leaf economic spectrum”, a fundamental trade-off between leaf traits associated with resource acquisition or conservation revealed by ecological studies [15]. The leaf economic spectrum has revealed two contrasted plant ecological strategies, acquisitive to conservative strategies, showing that a plant cannot maximise both the relative growth rate and nutrient retention [16]. A gradient of variation in trait values between resource acquisitive and conservative strategies also exists for roots, i.e. the so-called “root economic spectrum” [17,18]. In both leaf and root economic spectra, species with an acquisitive strategy are defined as making rapid use of nutrients, resulting in a high relative growth rate. Aboveground, they are characterised by a high specific leaf area (leaf area per biomass unit) and a low leaf dry-matter content [15] and, belowground, by a high specific root length (root length per biomass unit) and a small root diameter [17–19]. Conversely, species with a conservative strategy are defined as conserving nutrients, resulting in a low relative growth rate. They are characterised by the opposite combination of trait values. We thus assumed that assessing plant traits in order to characterize the resource exploitation strategies of a range of plant species would provide a basis for assessing their potential ability to compete for resources.

In a previous study [20], we hypothesised that traits related to exploration of an aboveground niche (e.g. plant height and width) and those related to light acquisition capacity (e.g. aboveground dry biomass) reflected plant light exploitation strategies. We assumed that characterising the light exploitation strategy of a range of plant species could generate information on their ability to compete for light and, therefore, to control weeds. On this basis, the light exploitation strategy was characterized in the French West Indies for 21 species, including potential cover plant species, weed species and banana cultivars. In line with this previous paper on light resources, the present study was focused on soil resource exploitation strategies. The overall objective was to characterize the soil exploitation strategies of these species using a trait-based approach, and thus to identify cover plant species with good potential to favour weed control via soil resource competition in banana cropping systems.

Plant root systems may have different architectures, which dictates their ability to grow depthwise and widthwise and to produce many roots. Plants thus have different soil exploration capacities [21]. Spatiotemporal soil exploration is known to play a key role in a plant’s access to both mobile resources, such as water and nitrogen, and relatively immobile resources, such as phosphorus ([22] and references therein, [23–27]). The ability of a plant species to explore a soil can be assessed both by the size of the zone explored by its root system and by the total root-impact density within the zone, representing the root occupation within this zone (Table 1). Besides, the ability of a plant to acquire soil resources within this soil zone can be assessed through its capacity to take up soil resources and transport them efficiently. Two root economic spectrum traits reflect these processes (Table 1): specific root length (with a high value reflecting an acquisitive strategy) and root diameter (with a high value reflecting a conservative strategy).

**Table 1:** Root traits used to characterize the ability of a plant species to exploit soil resources.

	Abbreviation	Description	Unit	Associated function	References	Mean (CV)
Traits related to spatial exploration of soil niches	Z	Median zone explored by the roots^1^	cm^2^	Soil interception zone (+)	[28]	764.8 (0.6)
	DI	Total root-impact density^2^	Number of root intersections.dm^-2^ of soil	Interception efficiency (+)	[10]	8.3 (0.6)
Traits related to resource acquisition	SRL	Specific root length	m.g^-1^ of root	Uptake capacity (+)	[29,30]	33.5 (0.9)
	D	Root diameter	Mm	Transport efficiency (+)	[17–19]	0.9 (0.8)
Markers of competition	DI 0–20 cm	Root-impact density in the 0–20 cm soil layer	Number of root intersections.dm^-2^ of soil	Niche complementarity		
	DI 40–80 cm	Root-impact density in the 40–80 cm soil layer				

^(+)^ indicates a positive relationship between the trait value and the function. CV = coefficient of variation corresponding to the mean/standard deviation.

^1^ The median zone explored by the roots was calculated as the product of the root depth and root width measured in a vertical soil profile.

^2^ Total root impact density was the number of root intersections divided the median zone explored by the roots.

Using the same species panel as in our previous study [20], the present paper characterised traits linked to the exploitation of soil resources (i.e. soil exploration by the root system and resource acquisition within the explored soil zone). Our aim was fourfold: (i) to identify root traits that differentiate plant species, (ii) to analyse correlations among root traits in order to determine whether soil-resource exploitation strategies could be identified in our panel of plant species, (iii) to analyse links between plant exploitation for soil resources (using data from the present study) vs. plant exploitation for light resources (using data presented in [20]), and (iv) to illustrate how the information gained in the present study could be used to facilitate the choice of potential cover plant species in cropping systems. The study was based on a 6-month field experiment, which is the maximum cycle duration of the annual cover crops studied, where plants were cultivated individually in classical soil and climate conditions for banana production.

## Materials and methods

### Plant species and experimental conditions

A field experiment was conducted to measure traits related to the exploration of both aboveground and belowground spatial niches and resource acquisition in 17 cover plant species and two weed species commonly found in banana cropping systems (Table 2 and S1 File). We also characterised two dessert banana cultivars: Cavendish, the cultivar grown in banana plantations worldwide, and a new cultivar created by CIRAD (cultivar 925), which is resistant to black Sigatoka (*Mycosphaerella fijiensis*), a major banana disease in the French West Indies. The experiment was conducted at the CIRAD Neufchateau experimental station in Guadeloupe (French West Indies, 16°04’48”N, 61°35’53”W, 263 m a.s.l.) from 24 April to 6 November 2013. It was carried out in a 0.4-ha field previously under fallow whose vegetation was destroyed with herbicides and spading before the beginning of the experiment. Four individual plants per species, corresponding to four repetitions, were sown by hand with a 16-m^2^ plot per plant (for *B*. *decumbens*, only three repetitions could have been processed). The plots were randomly distributed in the field. The soil was an andosol (FAO World reference base for soil resources). at the beginning of the experiment, the soil contained, 59.4 g organic matter kg^-1^, 34.4 g C kg^-1^, 3.5 g N kg^-1^ and 0.6 g P kg^-1^ on average, with a pH of 5.6. Fifty grams of urea fertilizer (46% of nitrogen) were applied at the base of each plant at the beginning of the experiment to ensure non-limiting nitrogen nutrition. Weeds that grew spontaneously around the plants were regularly removed by hand to ensure non-limiting growth conditions and to assess the growth potential of the tested species. Plants were grown under a rainfed regime with cumulated precipitation of 2,829 mm. The mean daily temperature was 25.6°C, ranging from 22.8°C to 29.7°C. Mean total solar radiation was 462 +/- 40 MJ.m^-2^.month^-1^.

**Table 2:** Species name, abbreviation and status in the banana cropping system. See photos of these plants in S1 File.

Species name	Abbreviation	Status	Plant type
*Arachis pintoï*	AP	Cover plant	Perennial herbaceous plant
*Brachiaria decumbens*	BD	Cover plant	Perennial grass
*Bidens pilosa*	BP	Weed	Annual herbaceous plant
*Brachiaria ruziziensis*	BR	Cover plant	Perennial grass
*Cajanus cajun* var. Guadeloupe	CCG	Cover plant	Pluri-annual shrub
*Centrosema pascuorum*	CP	Cover plant	Annual herbaceous plant
*Crotalaria spectabilis*	CS	Cover plant	Annual herbaceous plant
*Crotalaria zanzibarica*	CZ	Cover plant	Pluri-annual herbaceous plant
*Dolichos lablab*	DL	Cover plant	Pluri-annual vine
*Gliricidia sepium*	GS	Cover plant	Perennial tree
*Momordica charantia*	MC	Weed	Annual vine
*Mucuna pruriens* var. deeringiana	MD	Cover plant	Annual vine
*Musa spp*. var. CIRAD925	B925	Banana cultivar	Perennial herbaceous plant
*Musa spp*. var. Cavendish	Bcav	Banana cultivar	Perennial herbaceous plant
*Vigna unguiculata* var. David	N	Cover plant	Short annual vine
*Neonotonia wightii*	NW	Cover plant	Perennial vine
*Paspalum notatum*	PN	Cover plant	Perennial grass
*Pueraria phaseolides*	PP	Cover plant	Perennial vine
*Ricinus communis*	RC	Cover plant	Perennial shrub
*Stylosanthes guianensis*	SG	Cover plant	Pluri-annual
*Tagetes patula*	TP	Cover plant	Short annual herbaceous plant

### Plant traits related to belowground water and nutrient competition

Plant species were characterised with traits that accounted for soil exploration and resource acquisition. The traits are listed in Table 1 along with the main associated functions. We considered the four traits listed in the Introduction: the median zone explored by the roots (Z), total root-impact density (DI), specific root length (SRL) and root diameter (D). In addition, we described root distribution in the spatial niche with two markers, i.e. the DI values in two soil layers (0–20 cm and 40–80 cm).

### Measured and calculated plant traits

Aboveground traits [20] were measured as a complement to belowground traits to study possible correlations between aboveground and belowground traits and strategies. In species with a short life cycle, traits were measured at mid-flowering, i.e. when half the twigs had started to flower; in species with longer cycles, they were measured 6 months after sowing.

For belowground traits, a 1-m deep and 1-m wide trench was dug 20 cm from the base of the plant. The root intersections in the vertical soil profile were counted on a 5 cm × 5 cm mesh. The median root depth was calculated as the depth at which 50% of the root intersections were visible, and the median root width as the width at which 50% of the root intersections were visible. The median zone explored by the roots (Z) was calculated as the product of the median depth and median width. Median values were chosen to describe the zone where half of the roots—and presumably half of the acquisition—were present irrespective of the form of the root system. The total root-impact density (DI) was calculated as the number of visible root intersections divided by the surface area of the soil explored by the roots in a vertical soil profile. Like DI, the root-impact density per soil layer was calculated independently for the two soil layers (0–20 and 40–80 cm). For instance, in the 0–20 cm layer, DI was the number of visible root intersections between 0 and 20 cm soil depth divided by the surface area of the soil explored by the roots between 0 and 20 cm. For each cover plant species, the root-impact density was expressed relative to that of the Cavendish banana cultivar (Bcav) to assess the complementarity of the spatial niche in the two species. In the 0–20 soil layer: Δ0–20 = DI_*Bcav 0–20*_ –DI_*Cover plant 0–20*_. In the 40–80 soil layer: Δ 40–80 = DI _*Bcav 40–80*_ *–*DI _*Cover plant 40–80*_. The root-impact densities were analysed (i) in the 0–20 cm layer, where resources were plentiful due to fertilisation and (ii) in the 40–80 cm layer where nitrates were leached by rain.

In addition, for each plant, three 1000-cm^3^ soil samples were removed in the form of 10*10*10 cm cubes. The first sample was taken at the base of the plant at a depth of 0 to 10 cm, the second sample was taken under the base of the plant at a depth corresponding to half the maximum depth, and the third sample was taken at half the maximum width and depth. Each sample was washed to collect fine and coarse roots. The roots were scanned at 400 dots per inch (Epson expression 10000XL Pro scanner). The length and diameter of each root sample were measured with WinRHIZO Pro 2009a software (Regent Instruments, Quebec, Canada). The root diameter (D) was calculated by averaging the root diameters in three cubes per plant. The root contents of the three soil cubes were then pooled and weighed after drying for 72 h at 70°C to evaluate root dry biomass. The specific root length (SRL) was calculated by dividing the total length of the roots in the three samples by their root dry biomass.

For aboveground traits, the specific leaf area (SLA) was measured on three green leaves taken from the top of the plants using standard protocols [31]. The whole plant was used to measure aboveground dry biomass (Bma) and leaf dry biomass. In addition to Bma and SLA, the aboveground traits used to describe the competition abilities for light exploitation were height (H), crown width (CW) and leaf area ratio (LAR), with the latter being the ratio of total plant leaf area (product of leaf biomass and SLA) to Bma. The aboveground trait measurements are described in detail in [20].

### Statistical analysis

All statistical analyses were performed with R 3.1.1 software (alpha = 0.05) [32]. The Fligner test was conducted to assess the homogeneity in the variance of traits among species. The Kruskal-Wallis test (non-parametric test) was used to test differences between species due to the small number of replicates; we used the “Kruskal” function in the “*agricolae*” package [33] with false discovery rate correction. Correlations between traits were assessed using Spearman’s rank order correlation coefficient. Principal component analyses (PCA) were performed with the traits that explained most of the variability between species on the two first axes (with four replicates per species, except for two species with only three replicates, n = 82).

## Results

### Soil-resource exploitation traits

Mean values and coefficients of variation of functional traits across species are listed in Table 1 and the details for each species in the panel are listed in S1 Table. Traits exhibited marked variations among species. The trait with the largest (25-fold) difference among species was the median root exploration zone (Z) which ranged from 52 cm^2^ (*T*. *patula*) to 1323 cm^2^ (*P*. *phaseolides*). Root diameter (D) was the trait with the smallest (9-fold) difference among species, ranging from 0.22 (*B*. *pilosa*) to 1.99 mm (*D*. *lablab)*.

### Correlations between soil resource-exploitation traits

**Fig 1:**
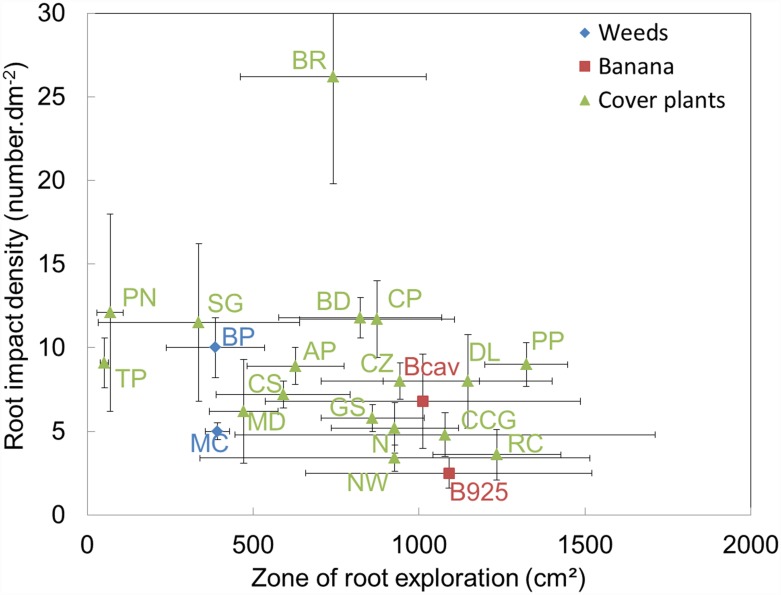
Species distributions regarding the relationship between total root-impact density and root exploration zone. See Table 2 for the species names. (n = 4 replicates for all species except BD (n = 3)).

**Fig 2:**
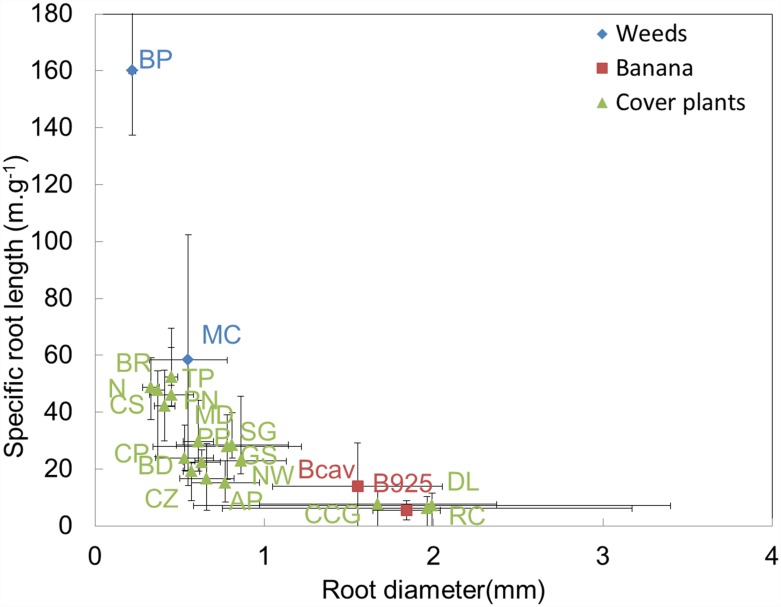
Species distributions regarding the relationship between specific root length and root diameter. See Table 2 for the species names. (n = 4 replicates for all species except BD (n = 3)).

Z was positively correlated with D and negatively correlated with the total root-impact density (DI) and specific root length (SRL) (Table 3 and Fig 1). D was negatively correlated with DI and SRL (Fig 2). DI and SRL were positively correlated.

**Table 3:** Correlations between root traits related to the ability of the species to exploit soil resources.

	Median zone explored by the roots (Z)	Total root-impact density (DI)	Specific root length (SRL)	Root diameter (D)
Median zone explored by the roots (Z)		**0.007**	**<0.001**	**<0.001**
Total root-impact density (DI)	**-0.30**		**<0.001**	**<0.001**
Specific root length (SRL)	**-0.54**	**0.39**		**<0.001**
Root diameter (D)	**0.45**	**-0.46**	**-0.89**	

The section below the diagonal presents the Spearman’s ranked correlation coefficient (n = 82). The section above the diagonal presents the P-value associated with the correlations. Significant correlations are in bold.

The weed species BP had a smaller Z and a higher DI than the banana cultivars (Fig 1). The cover plant species had a wide range of Z, and *B*. *ruziziensis* was unique as it had a very high DI and an intermediate Z.

As shown in Fig 2, the two weed species had smaller root diameters, D, than the banana cultivars and a much higher SRL. The roots of the cover plant species had intermediate D and SRL values compared to that of weed species and banana cultivars.

### Soil-resource exploitation strategies

**Fig 3:**
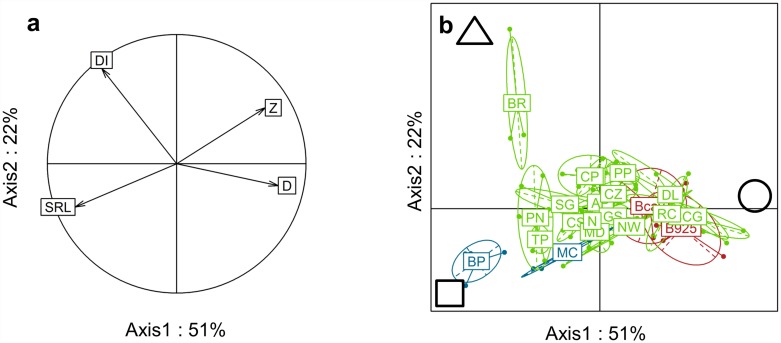
Principal component analysis on four soil-resource exploitation traits and 21 species (n = 4 replicates for all species except BD (n = 3)). **a** Correlation circle between traits; **b** Representation of weed species (in blue), banana cultivars (in red) and cover plant species (in green) on the first two axes (see Table 2 for the species names). The soil-resource exploitation traits are: SRL for specific root length, DI for total root-impact density, Z for median root exploration zone and D for root diameter. The symbols represent contrasted plant strategies, with a triangle for species with a high DI (total root-impact density), a square for species with a high SRL (specific root length) and a circle for species with high Z (median root exploration zone) and D (root diameter).

A principal component analysis of the 21 species, with four traits related to soil resource exploitation strategies, explained 73% of total variability on two axes (Fig 3a). Axes 1 and 2 explained 51% and 22% of variability, respectively. SRL, Z and D contributed (30%, 23% and 30%, respectively) to axis 1 while DI contributed (62%) to axis 2.

Axis 1 differentiates plant species with high D and high Z (on the right) from plant species with high SRL and high DI (on the left). Axis 2 differentiates plant species according to their DI values.

Fig 3b shows the projection of individual plants in the two-dimensional principal component analysis. Species were located along a continuum from high D and high Z (strategy represented by a circle in Fig 3b) to high SRL (strategy represented by a square in Fig 3b) or high DI (strategy represented by a triangle in Fig 3b). The two banana cultivars (Cavendish, Bcav; and Cirad 925, B925) were close and characterised by high Z, high D and low SRL. At the opposite end of the scale, the two weed species *B*. *pilosa* (BP) and *M*. *charantia* (MC) were characterised by high SRL and low Z values. The cover plant species were on a continuum from species with a high Z and high D (e.g. *C*. *cajan* var. Guadeloupe, CCG) to species with either a high SRL (e.g. *T*. *patula*, TP) or a high DI (e.g. *B*. *ruziziensis*, BR) (Fig 3b).

### Links between soil-resource and light exploitation traits

We characterised species for soil-resource exploitation traits (this study) and light exploitation traits [20]. We analysed correlations between the four traits explaining soil-resource exploitation and the five traits explaining light resource exploitation (Table 4). Among the correlations, Z was positively correlated with aboveground dry biomass (Bma), height (H) and crown width (CW). Bma was positively correlated with D and negatively correlated with SRL. H and CW were positively correlated with D and negatively correlated with SRL.

**Table 4:** Correlations between traits related to the ability to exploit soil resources (vertically) and traits related to the ability to exploit light (horizontally).

	Aboveground dry biomass (Bma)	Specific leaf area (SLA)	Leaf area ratio (LARa)	Height (H)	Plant crown width (CW)
Median root exploration zone (Z)	**0.63 <0.001**	-0.07 0.536	-0.10 0.388	**0.42 <0.001**	**0.52 <0.001**
Total root-impact density (DI)	0.02 0.836	0.18 0.154	-0.06 0.610	**-0.23 0.036**	-0.04 0.747
Specific root length (SRL)	**-0.55 <0.001**	0.19 0.085	0.16 0.137	**-0.45 <0.001**	**-0.34 <0.01**
Root diameter (D)	**0.47 <0.001**	**-0.26 0.020**	-0.16 0.153	**0.47 <0.001**	**0.27 0.013**

The first number in each box is the Spearman’s ranked correlation coefficient (n = 82); the second value is the P-value associated with the correlations; significant correlations (P<0.05) are in bold.

**Fig 4:**
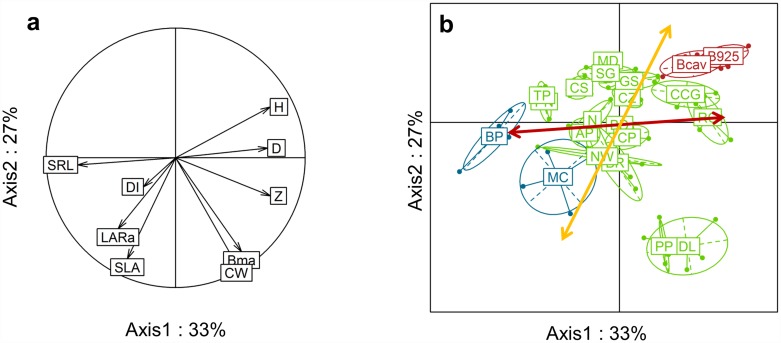
Principal component analysis on four soil-resource exploitation traits and six light exploitation traits and 21 species. **a** Correlation circle between traits; **b** Representation of weed species (in blue), banana cultivars (in red) and cover plant species (in green) on the first two axes (see Table 2 for the species names). The light exploitation traits are: Bma for aboveground dry biomass, SLA for specific leaf area, LARa for aboveground leaf area ratio, H for height and CW for plant crown width. The soil-resource exploitation traits are: SRL for specific root length, DI for total root-impact density, Z for the root exploration zone and D for root diameter. The yellow arrow represents the leaf economic spectrum gradient (according to the SLA direction) and the red arrow represents the root economic spectrum gradient (according to the SRL direction).

To analyse links between strategies for the exploitation of soil resources on the one hand and light on the other, PCA was performed on the 21 species with the four traits explaining soil-resource exploitation and the five traits explaining light exploitation (Fig 4). PCA showed that two axes explained 60% of total variability (Fig 4a). Axes 1 and 2 explained 33% and 27% of the variability, respectively. SRL, Z, D and H contributed significantly (19%, 18%, 17% and 18%, respectively) to axis 1. SLA, Bma, LARa and CW contributed significantly (25%, 22%, 12% and 28%, respectively) to axis 2.

Axis 1 mainly revealed a contrast between soil-resource exploitation, ranging from species with high D, high H and high Z (on the right) to species with high SRL (on the left). Axis 2 mainly differentiated plant species according to their light exploitation traits with high SLA and LARa values at the bottom and low values at the top.

Fig 4b shows projections of the two-dimensional PCA for individual plants. Species were located along a continuum from high D, high Z and low SLA (the two banana cultivars Bcav and B925), to high SRL, high SLA and high LARa, (the two weed species, *B*. *pilosa*–BP—and *M*. *charantia—*MC). The cover plant species were located between the two extremes and scattered according to Bma. Fig 4b shows the gradient in the leaf economic spectrum according to the SLA direction (acquisitive strategy marker; yellow arrow), and the gradient representing the root economic spectrum according to the direction of SRL and D (respectively markers of acquisitive and conservative strategies; red arrow). The two banana cultivars (Bcav and B925) displayed a conservative strategy for both light (low SLA) and soil resources (low SRL), while the two weed species displayed acquisitive strategies for both light (high SLA) and soil resources (high SRL). Some species, including *P*. *phaseolides* (PP) and *D*. *lablab* (DL), displayed a light acquisitive strategy and a soil-resource conservative strategy. Species such as *C*. *spectabilis* (CS) displayed a light conservative strategy and a soil-resource acquisitive strategy.

### Prospection zone for soil resources

**Fig 5:**
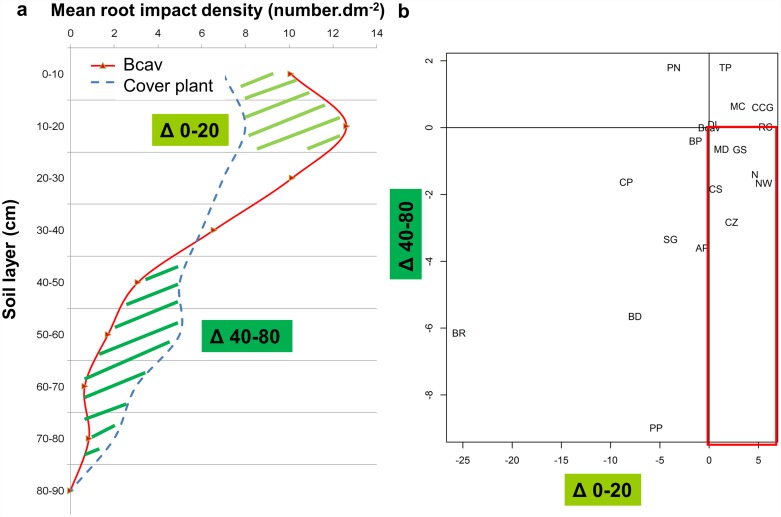
Species distribution according to differences in root-impact density between the cover plant species and the Cavendish banana cultivar (Bcav) in two soil layers (0–20 cm and 40–80 cm). **a** Banana root-impact density profile (red line) and root-impact density profile of an ideal cover plant species (blue dashed line) which is complementary to the banana profile, i.e. with a positive Δ0–20 (lower root-impact density than banana) and negative Δ40–80 (higher root-impact density than banana). **b** Species distribution according Δ0–20 and Δ40–80. The area outlined in red corresponds to the root-impact density profile required for associations with banana. See Table 2 for the species names.

The root-impact density revealed the soil occupation profile in each soil layer. The soil occupation profile of each cover plant and weed species was compared to that of the Cavendish banana cultivar (see example in Fig 5a). In two distinct soil layers (0–20 cm and 40–80 cm), a Δ was calculated for each cover plant species as the root impact density of banana minus the root impact density of the cover plant species. In the 0–20 cm soil layer, the root-impact density of species with a positive Δ0–20 was lower than that of banana, e.g. *T*. *patula* (TP) or *R*. *communis* (RC) in Fig 5b. In the 40–80 cm soil layer, the root-impact density of species with a negative Δ40–80 was higher than that of banana, e.g. *A*. *pintoï* (AP) or *B*. *ruziziensis* (BR) in Fig 5b. In banana, the roots that contributed most to soil-resource acquisition were located in the 0–20 cm soil layer. Consequently, species with a lower root density than banana in the 0–20 soil layer (positive Δ0–20 value) and a higher root density than banana in the deeper soil layer (negative Δ40–80) could be considered as species with a preferential root occupation zone complementary to the banana preferential zone, and therefore of potential interest for associations with banana. These species are at the bottom right in Fig 5b: *N*. *wightii*, *C*. *zanzibarica*, *C*. *spectabilis*, *V*. *unguiculata*, *M*. *deeringiana*, and *G*. *sepium* (NW, CZ, CS, N, MD and GS, respectively).

## Discussion

### Trade-offs between traits linked to spatial-niche exploration and soil-resource acquisition

We characterised a range of plant species according to their soil-resource exploitation strategies by assessing root traits involved in soil exploration by the root system and in the acquisition of resources within the explored soil zone. We observed trade-offs between traits.

In accordance with previous studies ([19] and references therein), we found a negative correlation between root diameter (D) and specific root length (SRL). This supports the hypothesis of a trade-off between processes involved in soil resource acquisition. Species with a high SRL are characterised by rapid nutrient acquisition and turn-over [17–19]. Large diameter roots have more mechanical strength to penetrate tough soil layers, which helps to access deeper soil resources while increasing the uptake of water and leached nutrients [34]. This is consistent with the root economic spectrum opposing conservation species (high D) and acquisition species (high SRL), regardless of botanical families [17,18].

Interestingly, we obtained a series of correlations showing that species exploring a large median soil zone (Z) had large diameter roots (D) but a low total root-impact density (DI). Conversely, species exploring a small soil zone had small diameter roots with a high total root-impact density. These findings suggest a trade-off between the species abilities to develop a limited number of large diameter roots exploring a large soil zone versus many thin roots limited to a smaller soil zone. They illustrated the scale versus precision foraging theory [29] opposing: (i) plant species that forage slowly for resources at a large scale but with limited spatial colonisation due to a small number of roots per soil unit (e.g. *R*. *communis*), and (ii) plant species that forage rapidly at a smaller scale but with more precision due to a large number of roots per soil unit, thereby increasing their chance of reaching nutrient patches (e.g. *S*. *guianensis*). These results are consistent with the two trade-offs found in the literature, i.e. a trade-off between the “exploitation potential” (Z in our study) and “exploitation efficiency” (DI in our study) shown in [28], and a trade-off of carbon allocation between producing thick roots (which are more “carbon-expensive” than thin roots) and fast growing roots [35].

### Plant species soil resource exploitation strategies

Characterising strategies of the panel of species with respect to soil resource exploitation revealed three contrasted strategies (Fig 3b), with species located along a continuum between these three strategies. (i) We identified species, such as banana, exploring a large soil volume (Z > 1,000 cm^2^) with large diameter roots (> 1.5 mm) able to penetrate tough soil layers (this strategy is represented by a circle in Fig 3b). As water is generally found in deep soil layers, plant species with this strategy may have the advantage of being able to acquire water and leached mobile resources. According to the root economic spectrum [17,18], these species could be considered as conservative. (ii) We identified species, such as *T*. *patula*, with a very high SRL (> 45 m.g^-1^) (this strategy is represented by a square in Fig 3b). The specific root length is known to be greatest in species with high nitrogen [36] and phosphorus [37] absorption abilities. Moreover, high SRL reflects a high relative growth rate [17,18,38]. We hypothesise that species with this strategy rapidly increase their root length to absorb nutrients efficiently. According to the root economic spectrum, these species could be considered as acquisitive. The strategies of both weed species were close to this strategy. Their rapid expected growth and nutrient acquisition could explain their abundance in the studied banana agroecosystem. (iii) We identified species, like *B*. *ruziziensis*, with a high total root-impact density (more than 11 root intersections.dm^-2^) and a small median soil zone (Z < 800 cm^2^) (this strategy is represented by a triangle in Fig 3b). We hypothesised that these species favour the production of many thin roots to explore the superficial soil layers. This strategy may favour the acquisition of immobile nutrients, such as phosphorus, in the uppermost soil layer [25,39,40].

### Links between aboveground and belowground traits and strategies

Until now, only a few papers have analysed the links between aboveground and belowground strategies using a range of plant species [41]. In our study, a few positive correlations were found between traits characterising the size of the aboveground and belowground spatial niches (Table 4): the median zone explored by roots (Z) was positively correlated with aboveground biomass (Bma), height (H) and crown width (CW). Although weak, a significant negative correlation was also found between the root diameter (a high D value is an indicator of a conservation strategy in the root economic spectrum) and the specific leaf area (a high SLA value is an indicator of an acquisition strategy in the leaf economic spectrum), in accordance with the findings of [42]. However, in contrast with [19], no significant correlation was found between the specific root length (SRL) and the specific leaf area (SLA), which are indicators of acquisitive strategies in the root and leaf economic spectra, respectively. Consequently, the gradient of the leaf economic spectrum according to the specific leaf area (SLA, marker of the acquisitive strategy; yellow arrow on Fig 4b) was not superimposed on the gradient representing the root economic spectrum positioned according to the specific root length and root diameter (SRL and D, respectively markers of acquisitive and conservative strategies; red arrow on Fig 4b). Overall, these results did not confirm the “plant economic spectrum” hypothesis put forward by Reich (2014), which presumes a similar strategy for aboveground and belowground compartments. Accordingly, some species displayed a similar ecological strategy aboveground and belowground—this was the case for banana cultivars with a conservative strategy for both light (low SLA) and soil resources (low SRL), and for the two weed species showing acquisitive strategies both aboveground and belowground. Moreover, some plant species (cover plant species in our study) displayed a light acquisitive strategy and a soil-resource conservative strategy, while others displayed a light conservative strategy and a soil-resource acquisitive strategy. Our findings thus indicated that all combinations between aboveground and belowground strategies are possible in plants.

### Practical application for identifying potential cover plant species to control weeds

**Fig 6:**
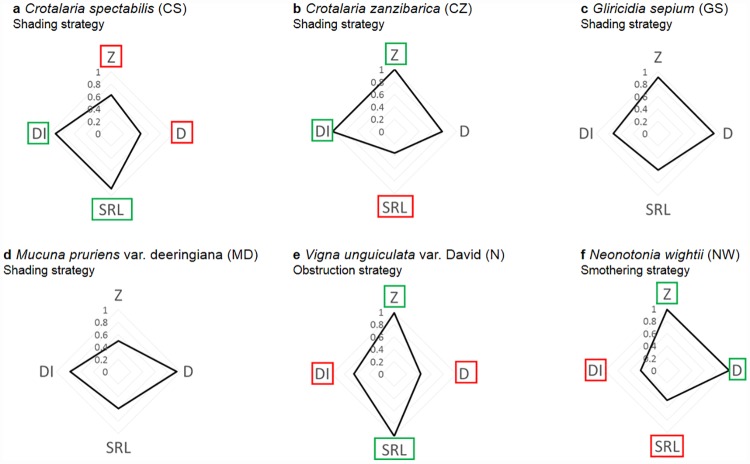
Comparison of the six potential cover crop species with regard to the four studied root traits: Root exploration zone (Z), root diameter (D), Specific Root Length (SRL) and total root impact Density (DI). **a** *Crotalaria spectabilis* (CS). **b** *Crotalaria zanzibarica* (CZ). **c** *Gliricidia sepium* (GS). **d** *Mucuna pruriens* var. *deeringiana*. **e** *Vigna unguiculata* var. David (N). **f** *Neonotonia wightii* (NW). For each trait, values are expressed in proportion to the highest value per trait observed for that trait in the panel of species studied. For each species, when the name of a trait is surrounded by green, then the species is one of the two species (among the six species) with the highest values for this trait. For each species, when the name of a trait is surrounded by red, then the species is one of the two species (among the six species) with the lowest values for this trait. Light exploitation strategy [see 20] of each species is mentioned under its name.

We assessed the potential of cover crop species to compete with weeds and banana by growing a large panel of cover plant species in the absence of competition with banana. Our results provided insight for identifying cover plant species with a good potential for controlling weeds in banana cropping systems. The objective when choosing a cover plant species for associations with banana is to simultaneously minimize competition with banana while maximising competition with weeds. Cover plant species are thus required to preferentially explore different soil layers from those explored by banana and, within these layers, to maximise soil exploration and resource acquisition. We compared the root-impact densities of different cover plant species in shallow and deep soil layers relative to that of banana (cv. Cavendish). We identified six cover plant species that preferentially explored deep soil layers, while banana explored shallow soil layers (Fig 5b). These species were therefore be assumed to minimize soil resource competition with banana. We also characterized each species by four traits related to spatial exploration of soil niches and acquisition of resources within the explored soil zone. A cover plant species with a good weed control potential could be expected to combine high values for these four traits. However, due to trade-offs between traits, none of the strategies identified and none of the studied species displayed this characteristic (Fig 3b). Therefore, we could not determine which of the six species, identified as minimizing competition with banana, had the best potential for maximising competition with weeds. Further studies should be focused on determining whether it is better to choose a species with intermediary values for each root trait (*Gliricidia sepium* in Fig 6c), or a species with high values for some traits and low values for others (five other species in Fig 6a, 6b, 6e, 6f and 6g). Indeed, at the current state of knowledge, it remains unclear which traits, among the four studied root traits, have the greatest impact for maximising competition with weeds. Moreover, in line with our previous study [20], the six potential cover plant species displayed different light exploitation strategies (Fig 6). Therefore, the ability of the six species (differing in both root traits and light exploitation strategies) to maximise competition with the most problematic weed species will have to be assessed in the field. Growing each of the six potential cover crop species, in competition with banana and/or weeds, would also allow us to search for any modifications in their rooting patterns in response to competition.

## Conclusions

With increasing interest in trait-based approaches to help in identifying potential cover plant species [10,43], the present study offers a new test of this approach. Of particular interest is the focus on root traits, an important yet understudied aspect of plant functional trait research. Our study generated answers to the four questions outlined in the Introduction. (i) All of the studied root traits varied among species and were therefore identified as relevant for analysing interspecific differences. (ii) Correlations among root traits indicated trade-offs between traits linked to spatial-niche exploration and soil-resource acquisition. Interestingly, they allowed us to position the different species on a scale versus precision foraging gradient and, on this basis, to identify three contrasted soil-resource exploitation strategies among species. (iii) We did not confirm the hypothesis of a plant spectrum due to the discrepancy between the belowground strategies (characterized in the present study) and the aboveground strategies (characterized in our previous paper [20]). (iv) The information gained in the present study illustrated how a trait-based approach could be used to identify potential cover plant species that could be introduced in cropping systems, according to their ability to compete for resources with weeds and banana. Among the 17 studied cover plant species, six were identified as potential good candidates. The next step will be to assess their performance in the field to biologically control weed species without affecting banana, in cropping systems with lower reliance on herbicides.

## Supporting information

S1 File Pictures of the 21 species/ and banana cultivars. (DOCX)

S1 Table Mean ± standard deviation for traits characterising soil resource exploitation. In each column, different letters indicate significant differences among species, letters were obtained with the Kruskal-Wallis post-hoc test (sample size n = 82, significant difference at P <0.05).
(DOCX)
