# Supplementary material for: Trait-based characterisation of soil exploitation strategies of banana, weeds and cover plant species
Source: PLoS One. 2017 Mar 3;12(3):e0173066. doi: 10.1371/journal.pone.0173066 (PMC5336259; doi:10.1371/journal.pone.0173066)
Supplement: S1 File — (DOCX) [file pone.0173066.s001.docx]

**S1 File. Pictures of the 21 species/ and banana cultivars.** Abbreviations used throughout the main text are indicated into brackets.


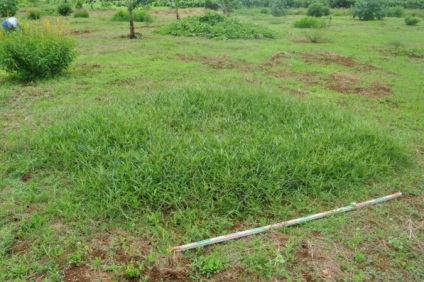


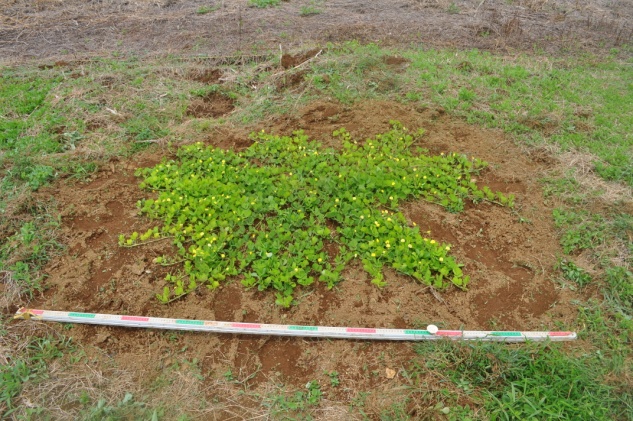


*Arachis pintoï* (AP) *Centrosema pascuorum* (CP)


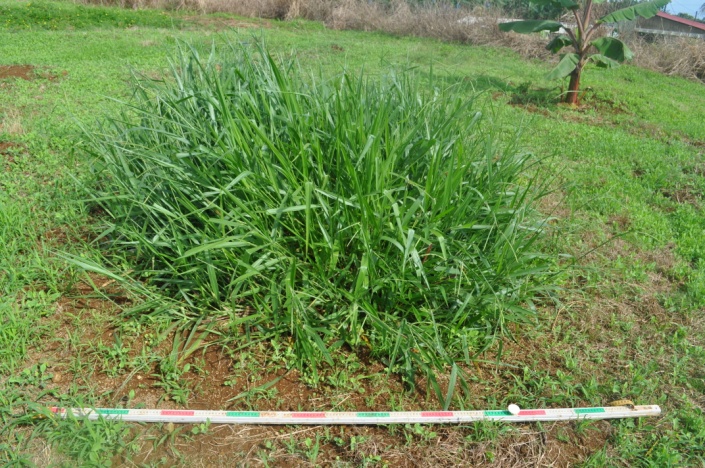

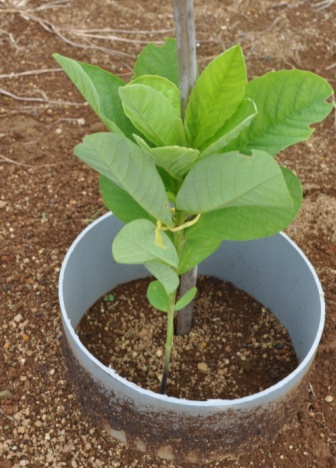


*Brachiaria decumbens* (BD) *Crotalaria spectabilis* (CS)


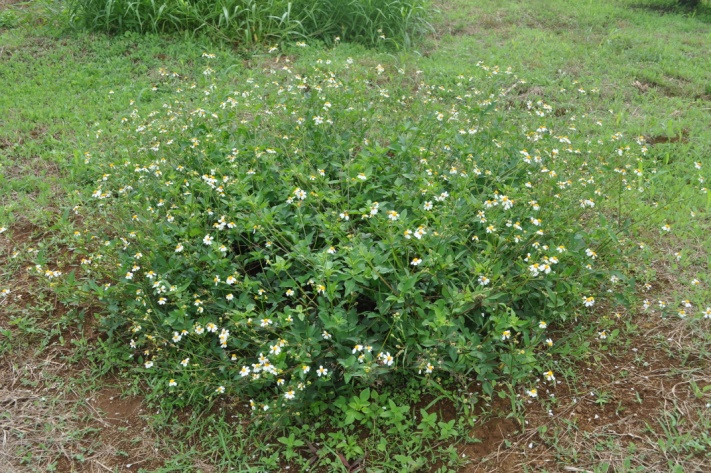

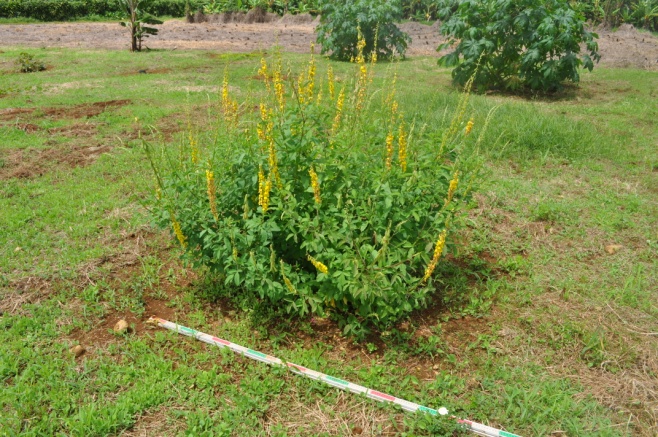


*Bidens pilosa* (BP) *Crotalaria zanzibarica* (CZ)


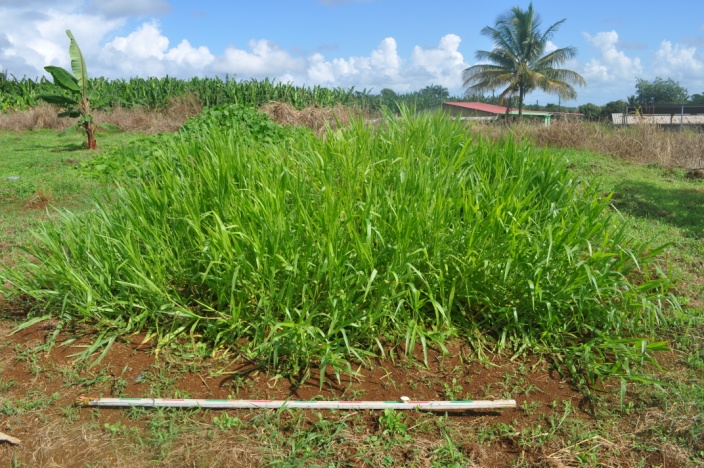

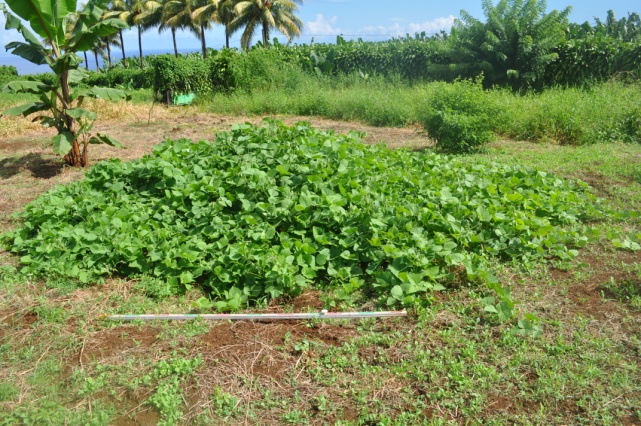


*Brachiaria ruziziensis* (BR) *Dolichos lablab* (DL)


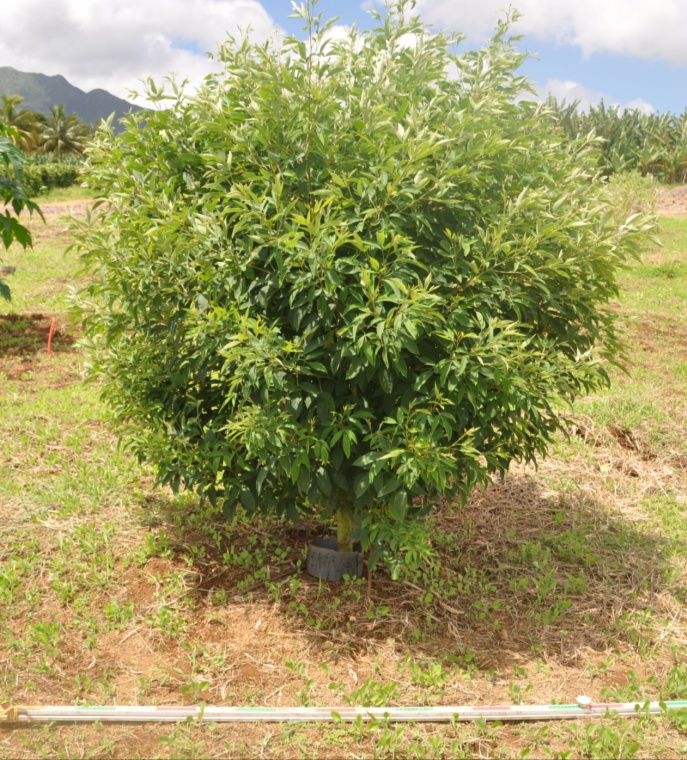

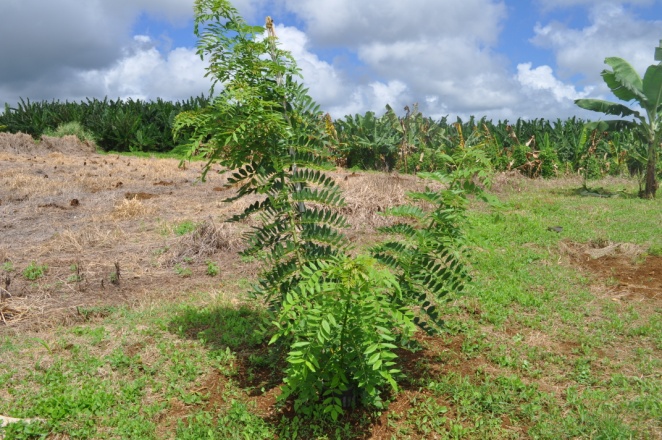


*Cajanus cajun* ‘Guadeloupe’ (CCG) *Gliricidia sepium* (GS)


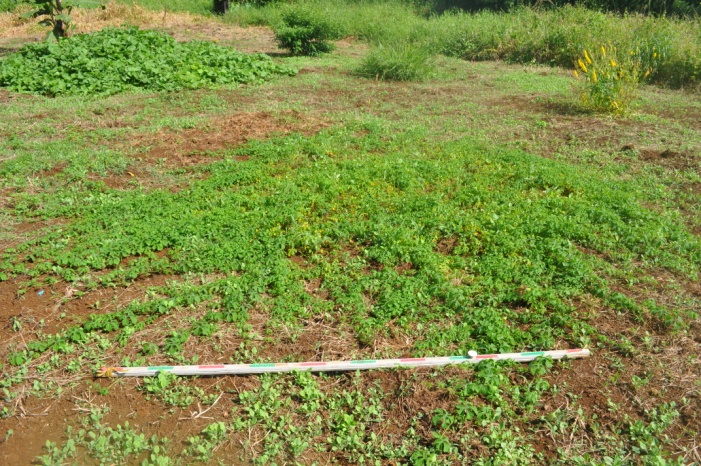

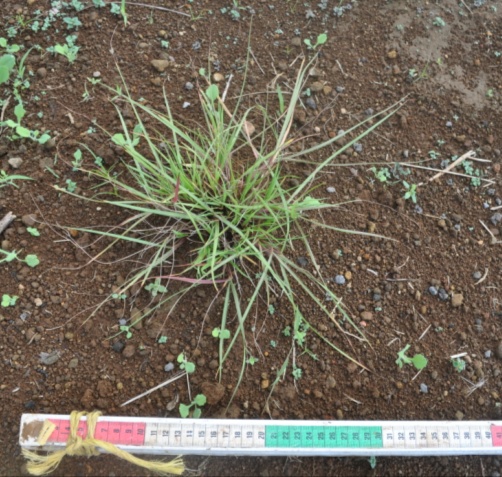


*Momordica charantia* (MC) *Paspalum notatum* (PN)


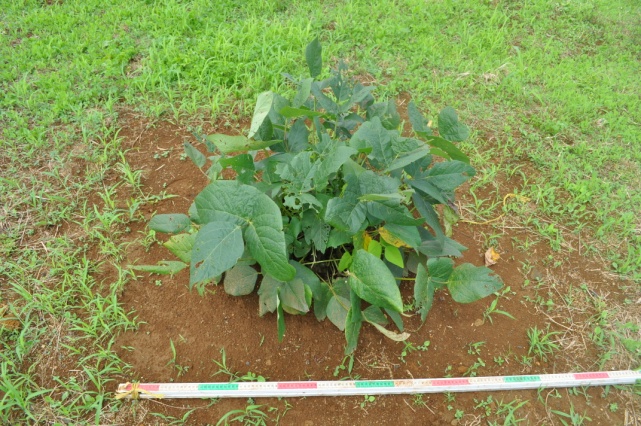

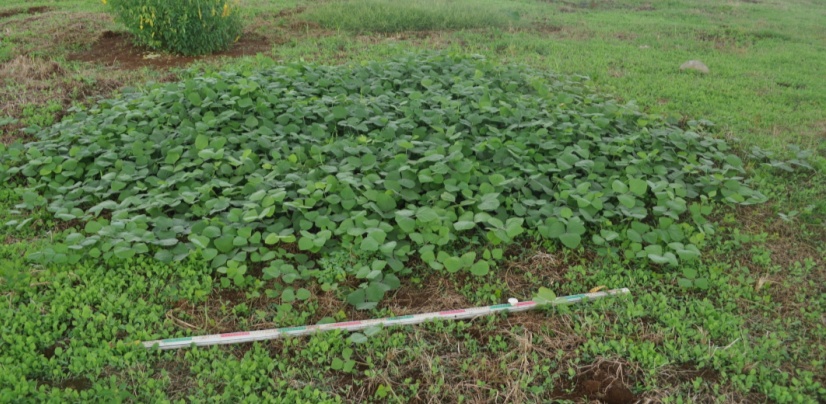


*Mucuna pruriens* var. deeringiana (MD) *Pueraria phaseolides* (PP)


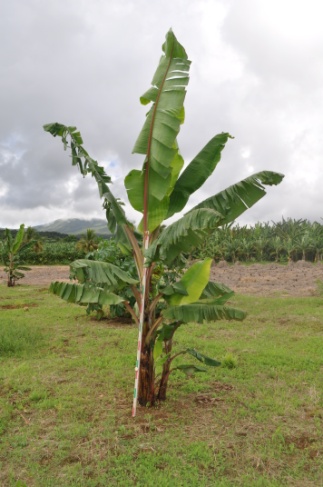

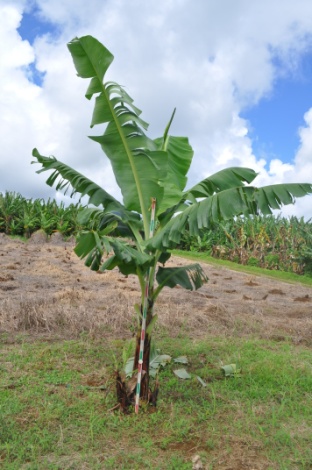

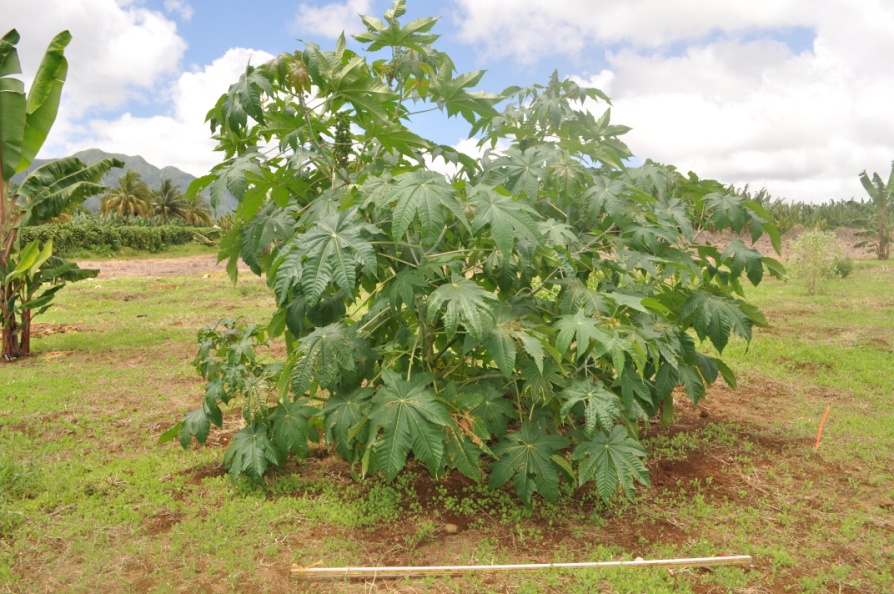


*Musa spp.*‘CIRAD925’ *Musa spp.* ‘Cavendish’ *Ricinus communis* (RC)

(B925) (Bcav)


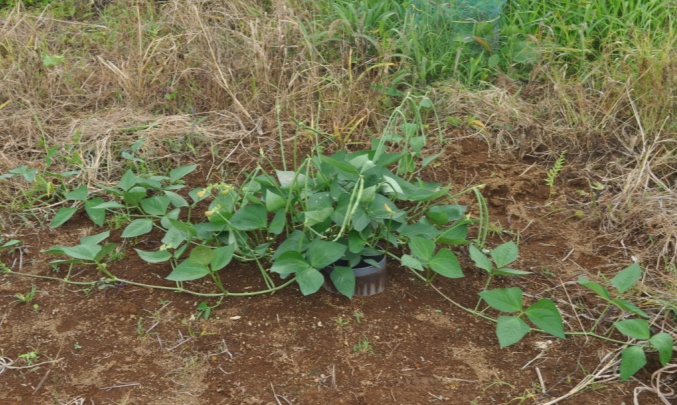

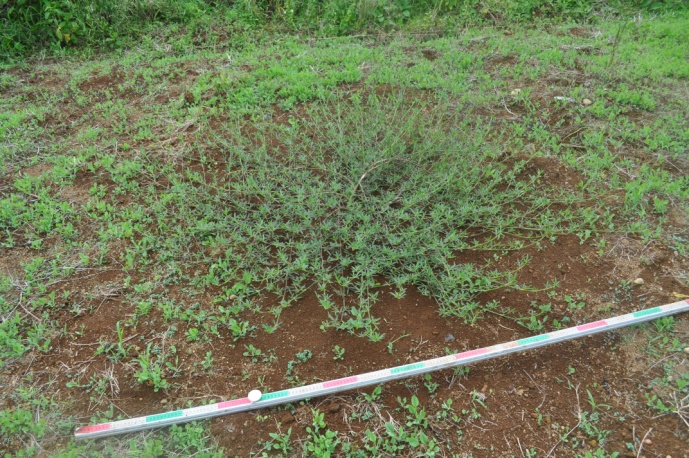


*Vigna unguiculata* var. David (N) *Stylosanthes guianensis* (SG)


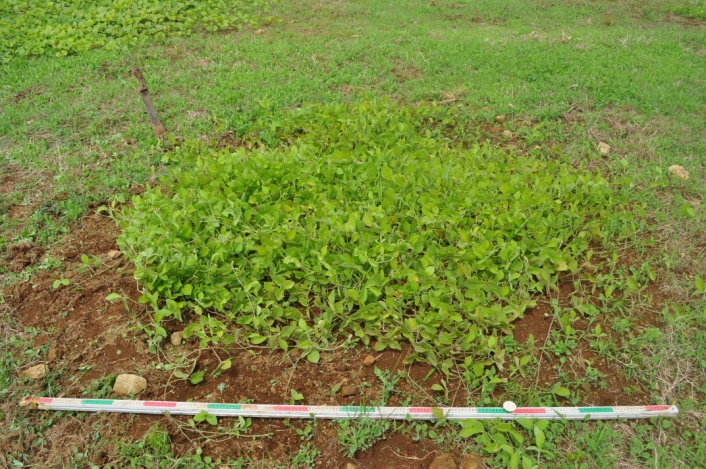

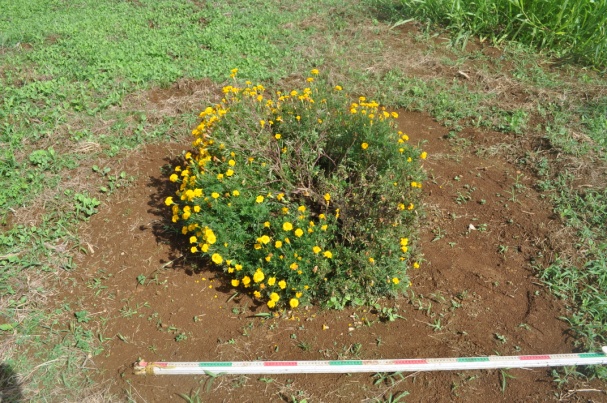


*Neonotonia wightii* (NW) *Tagetes patula* (TP)
