# Supplementary material for: Trait-based characterisation of soil exploitation strategies of banana, weeds and cover plant species
Source: PLoS One. 2017 Mar 3;12(3):e0173066. doi: 10.1371/journal.pone.0173066 (PMC5336259; doi:10.1371/journal.pone.0173066)
Supplement: S1 Table — In each column, different letters indicate significant differences among species, letters were obtained with the Kruskal-Wallis post-hoc test (sample size n = 82, significant difference at P <0.05). (DOCX) [file pone.0173066.s002.docx]

**S1 Table. Mean ± standard deviation for traits characterising soil resource exploitation.** In each column, different letters indicate significant differences among species, letters were obtained with the Kruskal-Wallis post-hoc test (sample size n=82, significant difference at P <0.05).

| Species name (abbreviation) | Specific root length  (SRL)  (m.g^-1^) | Zone explored by the roots  (Z)  (cm²) | Aboveground dry biomass  (BMa)  (kg) | Total leaf area  (TLA)  (cm²) | Root impact density in 0-20 cm soil layer  (DI 0-20)  (number.dm^-2^) | Root impact density in 40-80 cm soil layer  (DI 40-80)  (number.dm^-2^) | Root diameter (D)  (mm) | Root impact density on the whole profile  (DI)  (number.dm^-2^) |
| --- | --- | --- | --- | --- | --- | --- | --- | --- |
| *Arachis pintoï* (AP) | 15.3 ±7.68 fgh | 627 ± 146 defg | 0.51±0.23ef | 6.1 ± 2.3 f | 12 ± 2 bcde | 5 ± 4 abcd | 0.77 ± 0.2 c | 8,9 ± 1.1 bcd |
| *Musa spp.* ‘CIRAD925’ (B925) | 5.55 ± 3.37 h | 1090 ± 431 abc | 3.12±0.25c | 12.2 ± 0.8 e | 5 ± 2 h | 3 ± 5 efg | 1.84 ± 1.02 a | 2.5 ± 0.9 i |
| *Musa spp.* ‘Cavendish’ (Bcav) | 13.9 ± 15.2 gh | 1011 ± 475 abcd | 2.54±0.73c | 11.1 ± 3.0 e | 11 ± 6 cdef | 2 ±1 efg | 1.55 ± 0.39 a | 6.8 ± 2.8 def |
| *Brachiaria decumbens* (BD) | 19.3 ± 2.75 efg | 823 ± 246 bcde | 4.28±1.59c | 29.8 ± 7.8 cd | 19 ± 3 ab | 7±5 ab | 0.57 ± 0.05 cdef | 11.8 ± 1.2 ab |
| *Bidens pilosa* (BP) | 160 ± 22.6 a | 386 ± 148 gh | 0.04±0.02gh | 0,6 ± 0,2 gh | 13 ± 3 bcde | 2±3 efg | 0.22 ± 0.02 i | 10 ± 1.8 bc |
| *Brachiaria ruziziensis* (BR) | 48.8 ± 10.3 abc | 741 ± 280 cdef | 11.73±4.96ab | 43.5 ± 25.0 c | 37 ± 8 a | 8±5 ab | 0.33 ± 0.05 hi | 26.2 ± 6.4 a |
| *Cajanus cajun* ‘Guadeloupe’ (CCG) | 6.17 ± 4.17 h | 1234 ± 193 ab | 3.18±0.55c | 17.9 ± 6.8 de | 6 ± 4 fgh | 1±0 fgh | 1.96 ± 1.21 ab | 3.6 ± 1.5 ghi |
| *Centrosema pascuorum* (CP) | 24.0 ± 11.4 defg | 873 ± 234 bcde | 7.07±1.12b | 50.1 ± 7.5 bc | 20 ± 5 ab | 3±2 bcdef | 0.53 ± 0.17 defg | 11.7 ± 2.3 ab |
| *Crotalaria spectabilis* (CS) | 42.2 ± 12.5 bcd | 591 ± 202 efg | 0.12±0.07g | 1.0 ± 0.3 g | 11 ± 4 cdefgh | 4±2 abcde | 0.41 ± 0.06 fghi | 7.2 ± 0.8 def |
| *Crotalaria zanzibarica* (CZ) | 16.7 ± 12.2 fgh | 943 ± 239 abcde | 1.29±0.24d | 6.1± 1.4 f | 9 ± 2 defg | 5±1 abc | 0.66 ± 0.16 cd | 8 ± 1.1 cde |
| *Dolichos lablab* (DL) | 7.13 ± 4.54 h | 1147 ± 255 ab | 14.55±3.86a | 169.0 ± 35.7 ab | 11 ± 4 cdef | 2±1 efg | 1.99 ± 1.41 a | 8 ± 2,8 cde |
| *Gliricidia sepium* (GS) | 27.9 ± 11.2 cdef | 860 ± 155 bcde | 0.62±0.52ef | 4.3 ± 3.7 f | 8± 1 efgh | 2±2 cdefg | 0.78 ± 0.44 cd | 5.8 ± 0.8 efg |
| *Mucuna pruriens* var. deeringiana (MD) | 28.5 ± 11.4 bcdef | 471 ± 104 fgh | 0.37±0.19f | 0.8 ± 0.5 g | 10 ± 7 defg | 2±2 cdefg | 0.81 ± 0.33 c | 6.2 ± 3.1 ef |
| *Vigna unguiculata* var. David (N) | 47.8 ± 6.81 abc | 927 ± 192 abcd | 0.10±0.01g | 0.8 ± 0.3 g | 7 ± 5 fgh | 3±2 bcdefg | 0.37 ± 0.04 ghi | 5.2 ± 1.5 fgh |
| *Neonotonia wightii* (NW) | 22.8 ± 22.7 fg | 927 ± 588 bcde | 1.59±1.00d | 20.7 ± 12.3 de | 6 ± 4 fgh | 3±5 defg | 0.86 ± 0.27 bc | 3.4 ± 0.8 hi |
| *Momordica charantia* (MC) | 58.3 ± 44.1 bcde | 392 ± 36 gh | 1.35±1.21de | 24.1 ± 27.5 de | 9 ± 1 defgh | 1±1 gh | 0.55 ± 0.23 defg | 5 ± 0.5 fghi |
| *Paspalum notatum* (PN) | 46.2 ± 23.4 abcd | 69 ± 40 h | 0.05±0.04gh | 0.9 ± 0.7 g | 15 ± 9 bcde | 0±0 h | 0.45 ± 0.13 efgh | 12.1 ± 5.9 bc |
| *Pueraria phaseolides* (PP) | 22.5 ± 4.38 defg | 1323 ± 124 a | 16.32±5.03a | 182.1 ± 45.0 a | 17 ± 4 abc | 11±3 a | 0.63 ± 0.11 cde | 9 ± 1.3 bcd |
| *Ricinus communis* (RC) | 7.57 ± 4.61 h | 1078 ± 634 abcd | 8.39±1.99ab | 39.3 ± 11.9 c | 6 ± 2 gh | 2±1 efg | 1.67 ± 0.7 a | 4.8 ± 1.3 fghi |
| *Stylosanthes guianensis* (SG) | 29.7 ± 14.4 bcdef | 336 ± 303 gh | 1.04±0.36de | 2.5 ± 1.1 f | 15 ± 7 bcd | 5±1 ab | 0.61 ± 0.09 cde | 11.5 ± 4.7 bc |
| *Tagetes Patula* (TP) | 52.4 ± 10.4 ab | 52 ± 12 h | 0.01±0.00h | 0.1 ± 0.0 h | 10 ± 1 cdef | 0±0 h | 0.45 ± 0.04 efgh | 9.1 ± 1.5 bcd |
